# Analyses of zebrafish and *Xenopus *oocyte maturation reveal conserved and diverged features of translational regulation of maternal cyclin B1 mRNA

**DOI:** 10.1186/1471-213X-9-7

**Published:** 2009-01-28

**Authors:** Yan Zhang, Michael D Sheets

**Affiliations:** 1 University of Wisconsin School of Medicine and Public Health, Department of Biomolecular Chemistry, 1300 University Avenue Madison, Madison, Wisconsin 53706, USA

## Abstract

**Background:**

Vertebrate development relies on the regulated translation of stored maternal mRNAs, but how these regulatory mechanisms may have evolved to control translational efficiency of individual mRNAs is poorly understood. We compared the translational regulation and polyadenylation of the cyclin B1 mRNA during zebrafish and *Xenopus *oocyte maturation. Polyadenylation and translational activation of cyclin B1 mRNA is well characterized during *Xenopus *oocyte maturation. Specifically, *Xenopus *cyclin B1 mRNA is polyadenylated and translationally activated during oocyte maturation by proteins that recognize the conserved AAUAAA hexanucleotide and U-rich Cytoplasmic Polyadenylation Elements (CPEs) within cyclin B1 mRNA's 3'**U**n**T**ranslated **R**egion (3'**UTR**).

**Results:**

The zebrafish cyclin B1 mRNA was polyadenylated during zebrafish oocyte maturation. Furthermore, the zebrafish cyclin B1 mRNA's 3'UTR was sufficient to stimulate translation of a reporter mRNA during zebrafish oocyte maturation. This stimulation required both AAUAAA and U-rich CPE-like sequences. However, in contrast to AAUAAA, the positions and sequences of the functionally defined CPEs were poorly conserved between *Xenopus *and zebrafish cyclin B1 mRNA 3'UTRs. To determine whether these differences were relevant to translation efficiency, we analyzed the translational activity of reporter mRNAs containing either the zebrafish or *Xenopus *cyclin B1 mRNA 3'UTRs during both zebrafish and *Xenopus *oocyte maturation. The zebrafish cyclin B1 3'UTR was quantitatively less effective at stimulating polyadenylation and translation compared to the *Xenopus *cyclin B1 3'UTR during both zebrafish and *Xenopus *oocyte maturation.

**Conclusion:**

Although the factors that regulate translation of maternal mRNAs are highly conserved, the target sequences and overall sequence architecture within the 3'UTR of the cyclin B1 mRNA have diverged to affect translational efficiency, perhaps to optimize levels of cyclin B1 protein required by these different species during their earliest embryonic cell divisions.

## Background

In metazoans, early embryonic development is governed entirely by post-transcriptional mechanisms [1,2]. During this period of development translational mechanisms dictate when and how efficiently each maternal mRNA is translated into protein [3,4]. Many insights into these mechanisms come from studies of oocytes and eggs from the frog *Xenopus laevis*. For example, mechanisms that control maternal mRNA translation during *Xenopus *oocyte maturation are well studied and involve the regulated addition of 3' poly (A) to stored mRNAs [5,6]. It is unclear, however, how conserved these mechanisms are at the level of individual mRNAs. An understanding of such conservation should provide insights into how differences in target mRNA sequences could affect translational regulation during maternally controlled development.

The specificity of maturation-specific polyadenylation of maternal mRNAs during *Xenopus *oocyte maturation is controlled by sequences within the 3'UTRs of these mRNAs called **c**ytoplasmic **p**olyadenylation **e**lements (CPEs). CPEs are binding sites for CPEB (**CPE b**inding protein) [6-9]. In *Xenopus *oocytes CPEB binds other proteins, such as maskin that repress the translation of CPE-containing mRNAs [10]. During oocyte maturation CPEB phosphorylation stimulates its association with proteins that promote mRNA polyadenylation and translation [10-12]. CPEB is highly conserved in vertebrates, suggesting that CPE-regulated polyadenylation and translational activation during development is a highly conserved mechanism. In addition, recent studies have reported that some *Xenopus *maternal mRNAs contain polyadenylation response elements (PRE) that direct CPEB independent polyadenylation [13,14], but it is unclear whether PRE type polyadenylation occurs in organisms other than *Xenopus *[15]. Therefore, aside from studies in *Xenopus*, the mechanisms that control mRNA polyadenylation and translation during development in other vertebrate organisms have not been explored.

In this report we examine translational regulation of the maternal cyclin B1 mRNA during zebrafish oocyte maturation. Zebrafish oocytes, eggs and embryos offer many advantages for studying the maternal stages of vertebrate development. For example, maternal genes that regulate development have been identified in genetic screens [16-18], and the proteins encoded by these genes have provided insights into early vertebrate embryogenesis [19-21]. However, the mechanisms that govern maternal gene expression in zebrafish oocytes, eggs and embryos have not been explored. We chose to focus on cyclin B1 mRNA because its regulated polyadenylation and translation have been studied extensively during *Xenopus *oocyte maturation, and CPE-regulated translation is best characterized during this process. Using microinjection of reporter mRNAs into both zebrafish and *Xenopus *oocytes, we found that although translational regulation of both species' cyclin B1 mRNA was qualitatively similar, it differed quantitatively in terms of efficiency of both polyadenylation and translation. These quantitative differences were likely due distinctions between the 3'UTRs of zebrafish and *Xenopus *cyclin B1 mRNAs. These findings suggest that CPE-sequences and/or other aspects of 3'UTR sequence architecture evolved between species to modulate translational efficiency of maternal mRNAs, even those mRNAs that encode highly conserved proteins.

## Results

### Zebrafish cyclin B1 mRNA was polyadenylated during oocyte maturation and early embryogenesis

**Figure 1:**
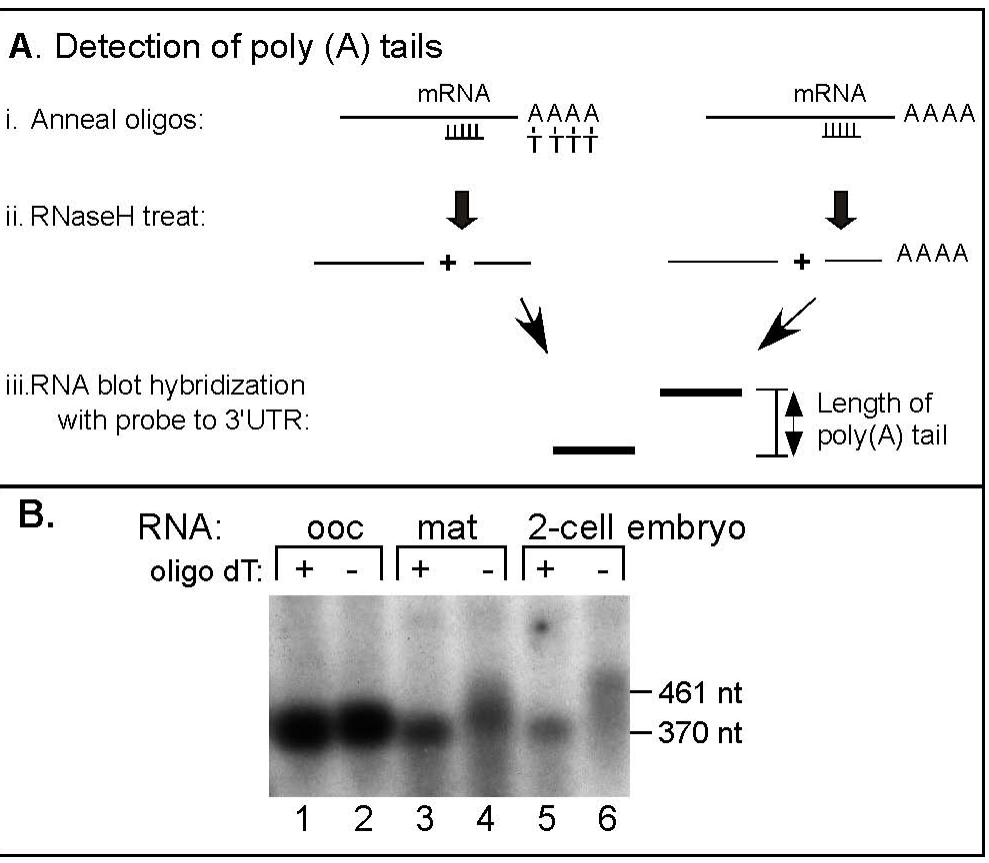
**The endogenous zebrafish cyclinB1 mRNA is polyadenylated during oocyte maturation and early embryogenesis**. (**A**) Diagram of oligonucleotide/RNaseH treatment for analysis of mRNA poly (A) tails. i.) RNA samples were hybridized to a DNA oligonucleotide complementary to a region of the open reading frame close to the 3'UTR. Half of each sample was also hybridized to oligo/dT. ii.) Treatment with RNaseH cleaved the RNA in the region of the RNA/DNA hybrid. In the sample with oligo/dT RNaseH cleaved the poly (A) tail. iii.) Treated RNA samples were analyzed by high resolution RNA blot hybridization and the sizes of the 3'UTR fragments with and without poly (A) indicates the length of poly (A) tail present. (**B**) Analysis of the endogenous zebrafish cyclinB1 mRNA 3'UTR following oligonucleotide/RNaseH treatment. Total RNAs from zebrafish oocytes, mature oocytes and two-cell embryos were hybridized with a DNA oligonucleotide complimentary to the zebrafish cyclin B1 mRNA's 3'UTR nucleotides -393 to -372 relative to poly(A) addition site at +1. Half of each sample was also hybridized with oligo dT (lanes 1, 3, 5). All samples were then treated with RNaseH and analyzed by RNA blot hybridization using a radio labeled probe corresponding to the 3'UTR of zebrafish CyclinB1 mRNA (-199 to +1). The positions of RNA markers are indicated.

Translation of the zebrafish cyclin B1 mRNA is activated during oocyte maturation but the underlying mechanisms have not been examined [22,23]. However, extensive analysis of *Xenopus *cyclin B1 mRNA has demonstrated that poly (A) addition regulates the translation of this mRNA during oocyte maturation. Therefore, we analyzed the polyadenylation state of the endogenous zebrafish cyclin B1 mRNA during zebrafish oocyte maturation. For this analysis RNaseH treatment of purified RNA samples was used to sever the 3'UTR from the cyclin B1 mRNA and facilitate the direct detection of a 3' poly (A) tail by RNA blot hybridization [24]. RNA isolated from oocytes, matured oocytes and two-cell embryos was hybridized to a DNA oligonucleotide complementary to the cyclin B1 ORF (372 nucleotides 5' of the poly (A) addition site) and half of each sample was also hybridized to oligo-dT to remove poly (A) (Fig. 1A). Each sample was treated with RNaseH and analyzed subsequently by high resolution RNA blot hybridization with a radiolabeled probe complementary to the zebrafish cyclin B1 3'UTR. The zebrafish cyclin B1 mRNA from oocytes either lacked or possessed only an extremely short poly (A) tail as the size of the 3'UTR was by unaffected by the addition of oligo dT (Fig. 1B, **lanes 1 and 2**). In contrast, the cyclin B1 mRNA from matured oocytes and 2-cell embryos possessed a poly (A) tail that ranged from 50 to 150 nucleotides in length (Fig. 1B. **lanes 3 to 6**). Similar results were obtained using RNA-ligated RT-PCR to measure poly (A) tails (data not shown) [14]. Therefore, the endogenous zebrafish cyclin B1 mRNA was polyadenylated during oocyte maturation coincident with its translational activation [23].

### Polyadenylation of the zebrafish cyclin B1 mRNA depended upon U-rich 3'UTR sequences

**Figure 2:**
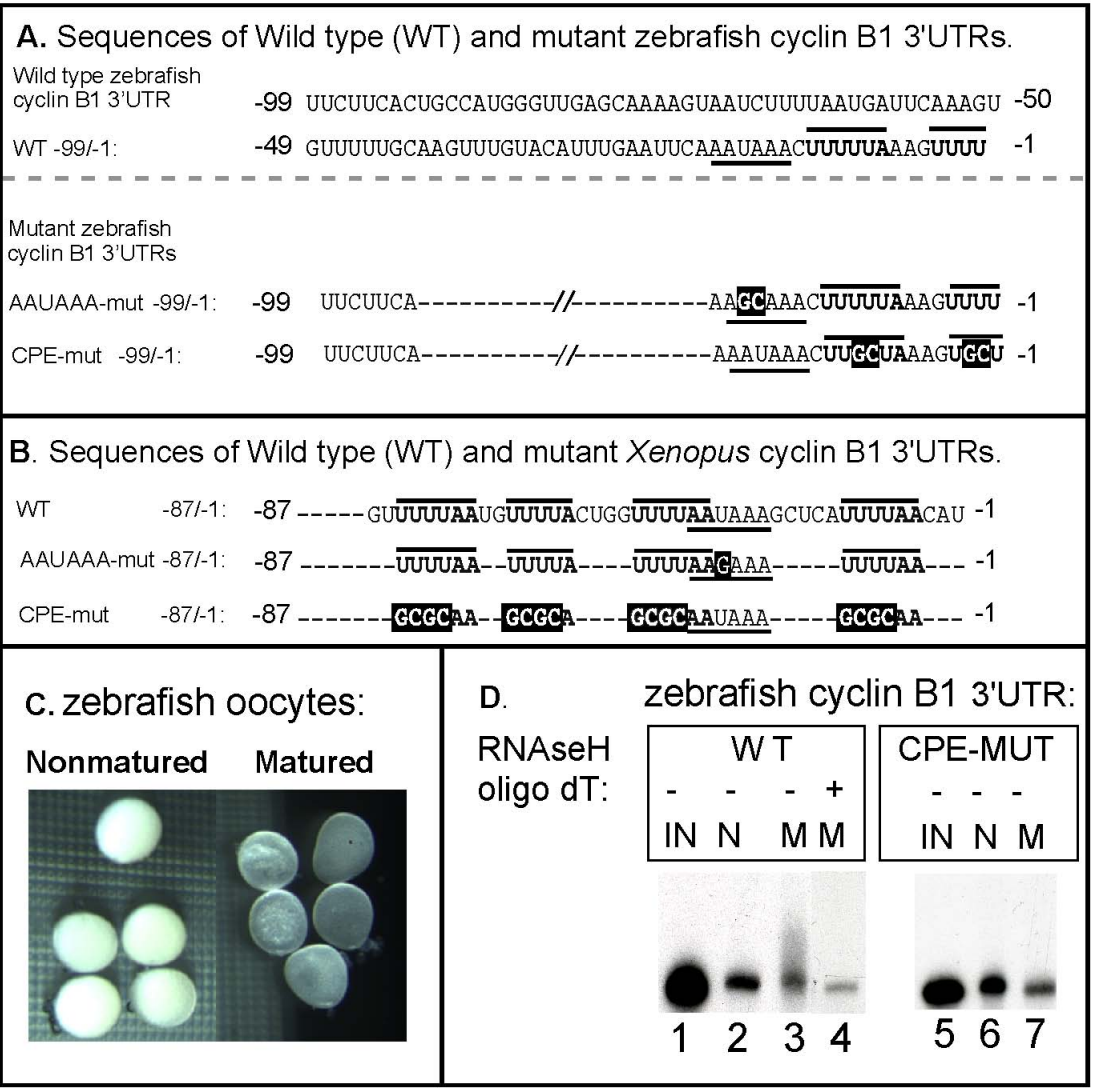
**Polyadenylation of the zebrafish cyclin B1 mRNA 3'UTR depended upon U-rich CPE sequences**. (**A**) Sequences of wild-type, AAUAAA mutant and U-rich mutant zebrafish cyclin B1 3'UTRs. The zebrafish cyclin B1 3'UTR is 199 nucleotides in length and the last 99 nucleotides are shown. U-rich putative CPE elements adjacent to AAUAAA are overlined and bolded while the AAUAAA sequence is underlined. Mutated sequences are white on black. Other U-rich sequences are present in the zebrafish cyclin B1 3'UTR, but their distance from AAUAAA makes it unlikely that they function as CPEs. (**B**) Sequences of wild-type, AAUAAA mutant and CPE mutant *Xenopus *cyclinB1 3'UTRs. Mutated sequences are white on black, CPE elements are overlined and bolded and AAUAAA elements are underlined. The numbers indicate positions of each nucleotide relative to the poly (A) addition site. (**C**) Zebrafish oocytes and matured oocytes. (**D**) Zebrafish cyclin B1 3'UTR is sufficient to direct polyadenylation during oocyte maturation, and the CPEs are required. P^32^-labeled RNA consisting of the zebrafish cyclin B1 mRNA 3'UTR was injected into 50–100 zebrafish oocytes and some oocytes were matured. RNA from injected non-mature oocytes (N) or injected matured oocytes (M) were analyzed by 4% denaturing PAGE and autoradiography. IN: Uninjected RNA. N: RNA from non-mature oocytes. M: RNA from mature oocytes. As controls, half of the total RNAs extracted from WT 3'UTR RNA injected matured oocytes were subjected to oligo dT/RNaseH treatment prior to denaturing PAGE (lane 4). The size reduction after oligo dT/RNaseH treatment indicated that the 3'UTR RNA was polyadenylated.

During *Xenopus *oocyte maturation, cytoplasmic polyadenylation of maternal mRNAs depends upon specific sequence elements within mRNA 3'UTRs: typically the CPE (cytoplasmic polyadenylation element) and the AAUAAA hexanucleotide [6,7,9,25,26]. A subset of *Xenopus *mRNAs are polyadenylated due to the presence of PRE (polyadenylation response elements) [13,14]. The vast majority of eukaryotic mRNAs contain the highly conserved AAUAAA hexanucleotide within their 3' UTRs. AAUAAA is required for formation of the primary mRNA transcript's mature 3' end, which involves both the cleavage and polyadenylation of the newly synthesized transcript in the nucleus [27,28]. AAUAAA is also required for the cytoplasmic polyadenylation that occurs on only a subset of maternal mRNAs during vertebrate development. This subset of mRNAs also contains CPEs. In contrast to AAUAAA, CPEs vary in terms of numbers, exact sequence and position within the 3'UTR. The canonical CPE sequence is UUUUAU, but the non-canonical sequences UUUUUCAU, UUUUAAU and UUUUACU can also direct cytoplasmic polyadenylation [26]. The *Xenopus *cyclin B1 3'UTR contains four CPEs close to the AAUAAA (Fig. 2B). Mutations affecting either the AAUAAA sequence or all four CPEs abolish maturation specific polyadenylation [7,9,24,29]. Mutation of any single CPE is not sufficient to abolish polyadenylation, suggesting some redundancy among these elements.

To help define putative CPEs in the zebrafish cyclin B1 3'UTR, we compared the cyclin B1 mRNA 3'UTRs from zebrafish and *Xenopus*. Although cyclin B1 protein is highly conserved, the cyclin B1 mRNA's 3'UTRs exhibit very limited sequence identity (data not shown). Nevertheless, the zebrafish cyclin B1 3'UTR contains two sequences of four contiguous U nucleotides positioned immediately 3' to AAUAAA. These characteristics, U-rich sequences close to AAUAAA are hallmarks of CPEs found in the *Xenopus *cyclin B1 3'UTR (Fig. 2B). There is another U-rich sequence (UUUUAAU, position -66/-60), but since CPE function decreases significantly as the distance from the AAUAAA sequence increases [26] we focused on potential U-rich sequences closest to the hexanucleotide. To test whether the U-rich sequences in the zebrafish cyclin B1 3'UTR functioned as CPEs, two radiolabeled 3'UTR RNAs (referred to as WT and CPE-MUT) were generated. The WT RNA contained the wild type 3'UTR and the CPE-Mut RNA contained UU to CG substitutions within each of the two putative CPEs in the zebrafish cyclin B1 3'UTR (Fig. 2A). Both radiolabeled RNAs were injected into zebrafish oocytes and a fraction of injected oocytes were exposed to 7α, 20β-dihydroxyprogesterone to induce maturation, while the remaining fraction was left untreated. Oocyte maturation was monitored based on translucence, a defining characteristic of mature zebrafish oocytes (Fig. 2C). After maturation was complete, RNA was isolated from injected oocytes and analyzed by denaturing gel electrophoresis. A portion of the injected WT cyclin B1 3'UTR RNA increased in size during oocyte maturation (Fig. 2D, **lanes 2 and 3**) due to the addition of a poly (A) as treatment with oligo-dT/RNaseH converted the high molecular weight species to the size of the injected RNA (Fig. 2D, **lane 4**). In contrast, the CPE-MUT cyclin B1 3'UTR RNA was not polyadenylated during oocyte maturation (Fig. 2D, **lane 5–7**). These results suggested that the zebrafish cyclin B1 3'UTR contained U-rich CPEs required for polyadenylation during oocyte maturation.

### The zebrafish cyclin B1 mRNA 3'UTR was sufficient to activate translation during oocyte maturation in a CPE- and AAUAAA-dependent manner

**Figure 3:**
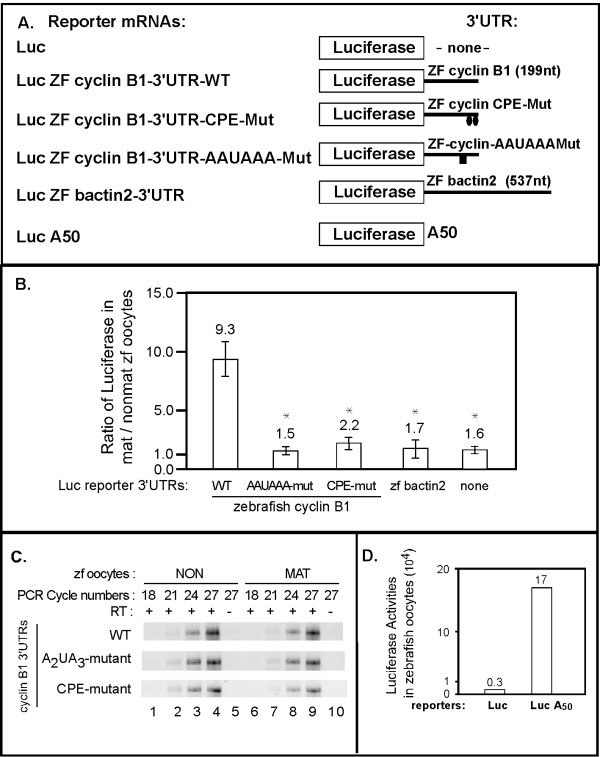
**The 3'UTR of zebrafish cyclinB1 mRNA is sufficient to activate translation during zebrafish oocyte maturation and this activation requires the CPE and AAUAAA sequences**. (**A**) Schematic of various luciferase reporter mRNAs with different 3'UTRs (ZF-zebrafish). (**B**) The zebrafish cyclin B1 3'UTR directs translational activation during oocyte maturation and activation requires the putative CPE elements and AAUAAA element. Luciferase reporter mRNAs with various zebrafish cyclinB1 3'UTRs were injected into oocytes. Some of the injected oocytes were matured. Luciferase activity was measured in extracts prepared from injected oocytes and injected matured oocytes. The ratio of luciferase in matured versus non-matured oocytes was plotted for comparison. The mat/non-mature ratios shown are represented as mean +/- SEM of at least three independent experiments. Statistical analysis (P values) was performed by one-way ANOVA. * P < 0.05. Each reporter containing zebrafish cyclin B1 mutant 3'UTR, bactin2 3'UTR or no 3'UTR was compared to the reporter with zebrafish cyclin B1 WT 3'UTR. (**C**) Luciferase reporters were equally stable in mature vs. non-mature oocytes. Total RNA was extracted from mature and non-matured oocytes injected with each zebrafish cyclin B1 3'UTR reporter mRNA. Equal amounts of total RNA from each sample were utilized in quantitative RT-PCR using luciferase-specific primers. Product formation was monitored with increasing cycles of amplification and analyzed by agarose gel electrophoresis. (**D**) The presence of a poly (A) tail is sufficient to stimulate mRNA translation in zebrafish oocytes. Luciferase reporter mRNAs (Luc vs. LucA50) were injected into 20–30 fully-grown zebrafish oocytes and assayed for luciferase activity after 6 hours. This experiment was repeated twice and shown here are the absolute values of luciferase activity from one representative experiment.

To analyze the role of the cyclin B1 3'UTR on translational activation, a luciferase reporter mRNA fused to the 3'UTR of the zebrafish cyclin B1 mRNA was generated (Luc-ZF cyclin B1-3'UTR-WT) (Fig. 3A). The wild type reporter mRNA (Luc-ZF-cyclin B1-3'UTR-WT) was injected into zebrafish oocytes that were subsequently treated as described above for Fig. 2. Extracts from the injected cells were assayed for luciferase activity. Changes in translational activity during oocyte maturation were expressed as the ratio of luciferase activity from matured oocytes to that from non-matured oocytes (Fig. 2B). Translation of the wild type reporter mRNA (luc-ZF-cyclin B1-3'UTR-WT) was stimulated almost 9 fold during zebrafish oocyte maturation (Fig. 3B). This stimulation was specific for the cyclin B1 3'UTR as translation of other reporter mRNAs that contained no 3'UTR (Luc), or the cytoskeletal b-actin 3'UTR were not stimulated significantly during oocyte maturation (Fig. 3B). Thus, the 3'UTR of the zebrafish cyclin B1 mRNA contained sequences that could activate translation during oocyte maturation.

To test whether the same sequences required for zebrafish cyclin B1 mRNA polyadenylation (Fig. 2) were also required for translational activation, luciferase reporter mRNAs were generated that contained the zebrafish cyclin B1 3'UTRs in which the two CPE (luc-ZF-cyclin B1-CPE-Mut) or the AAUAAA (luc-ZF-cyclin B1-AAUAAA-Mut) sequences were mutated (Fig. 3A and see Fig. 2A for specific mutations). Translational activity of these mutant reporter mRNAs was compared to the wild type reporter in zebrafish oocyte microinjection experiments as described above. Mutation of either the CPE sequences (luc-ZF-cyclin B1-CPE-Mut) or the AAUAAA (luc-ZF-cyclin B1-AAUAAA-Mut) that abolished zebrafish cyclin B1 mRNA polyadenylation (Fig. 2) significantly reduced the ability of the zebrafish cyclin B1 mRNA's 3'UTR to stimulate translation of the luciferase reporter mRNA during oocyte maturation (Fig. 3B). RT-PCR analysis demonstrated that the injected wild type, CPE-mutant and AAUAAA mutant reporter mRNAs were equally stable in mature and non-mature oocytes (Fig. 3C). Therefore, the zebrafish cyclin B1 3'UTR can activate mRNA translation during oocyte maturation and this activation depends upon the same CPE and AAUAAA sequences required for maturation-specific polyadenylation.

To directly assess the role of the poly (A) tail on translation in zebrafish oocytes, translation of two additional luciferase reporter mRNAs was measured in zebrafish oocytes. These reporters were identical except for the presence of a fifty-nucleotide 3' poly (A) tail. (Fig. 3A) [24,30]. Equal amounts of each mRNA were injected into separate groups of oocytes. The injected oocytes were cultured for six hours (these oocytes were not induced to mature) and then extracts prepared from these oocytes were assayed for luciferase activity. Translation of the reporter mRNA containing the poly (A) tail (LucA_50_) was significantly higher than that of the reporter mRNA lacking a poly (A) tail (Luc), indicating that presence of a poly (A) tail was sufficient to stimulate mRNA translation in zebrafish oocytes (Fig. 3D). Therefore, 3' poly (A) is sufficient to enhance translation in zebrafish oocytes.

### The zebrafish cyclin B1 3'UTR was less efficiently polyadenylated and less effective at enhancing translation during either zebrafish or *Xenopus oocyte *maturation

**Figure 4:**
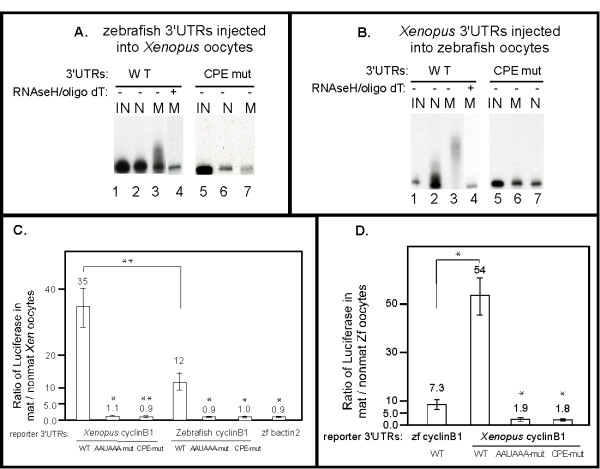
**Directing polyadenylation and activating translation during oocyte maturation are evolutionarily conserved functions of cyclin B1 3'UTRs**. (**A**) The zebrafish cyclinB1 3'UTR is sufficient to direct polyadenylation during *Xenopus *oocyte maturation, and the CPEs are required. Each P^32^-labeled 3'UTR RNA was injected into 30–50 *Xenopus *oocytes and some oocytes were matured. RNA isolated from injected non-mature oocytes or injected matured oocytes were analyzed by 4% denaturing PAGE. IN: Uninjected RNA. N: RNA from non-mature oocytes. M: RNA from mature oocytes. Half of the RNA from WT 3'UTR injected mature oocytes was treated with oligo dT/RNaseH prior to analysis (lane 4). The size reduction after oligo dT/RNaseH treatment (lane 4 versus lane 3) indicated that the WT 3'UTR RNA was polyadenylated. (**B**) The *Xenopus *cyclinB1 3'UTR is sufficient to direct polyadenylation during zebrafish oocyte maturation, and the CPEs are required. Each P^32^-labeled 3'UTR RNA was injected into 50–100 zebrafish oocytes and some oocytes were matured. RNA from injected non-matured oocytes or injected matured oocytes were analyzed by 4% denaturing PAGE. IN: Uninjected RNA. N: RNA from non-mature oocytes. M: RNA from mature oocytes. Half of the RNA extracted from WT 3'UTR injected matured oocytes was treated with oligo dT/RNaseH prior to analysis (lane 4). The size reduction after oligo dT/RNaseH treatment indicated that the WT 3'UTR RNA was polyadenylated (compare lanes 3 and 4). (**C**) The zebrafish cyclinB1 3'UTR is sufficient to activate translation during *Xenopus *oocyte maturation and the AAUAAA and CPE sequences are required for this activation. Luciferase reporter RNAs with various 3'UTRs were injected into 30–40 *Xenopus *oocytes and half were matured with progesterone. The ratios of luciferase activities from mature vs. non-mature oocytes were calculated and graphed as mean +/- SEM from at least three independent experiments. Statistical analysis was performed by one-way ANOVA. * P < 0.05, ** P < 0.01. Each *Xenopus *mutant 3'UTR was compared to *Xenopus *WT 3'UTR, each zebrafish mutant cyclin B1 3'UTR or WT bactin2 3'UTR was compared to zebrafish WT cyclin B1 3'UTR. The mature/non-mature ratio of *Xenopus *cyclin B1 3'UTR is significantly different from that of zebrafish cyclin B1 3'UTR (**D**) The *Xenopus *Cyclin B1 3'UTR is sufficient to activate translation during zebrafish oocyte maturation and the AAUAAA and CPE sequences are required for this activation. Luciferase reporter RNAs with various 3'UTRs were injected into 50–100 zebrafish oocytes and half were matured with hormone. The ratio of luciferase activities from mature versus non-mature oocytes were calculated and graphed as the mean values +/- SEM from at least three independent experiments. Statistical analysis was performed by one-way ANOVA. * P < 0.05. Each mutant *Xenopus *reporter was compared to WT *Xenopus *reporter. The mature/non-mature ratio of zebrafish cyclin B1 3'UTR is significantly different from that of *Xenopus *cyclin B1 3'UTR.

The 3'UTRs of the zebrafish and *Xenopus *cyclin B1 mRNAs share little sequence similarity aside from the presence of CPEs and the AAUAAA hexanucleotide elements (data not shown and Figs. 2A and 2B). We noted that zebrafish cyclin B1 mRNA appeared to be less efficiently polyadenylated in zebrafish oocytes than what has been observed for *Xenopus *cyclin B1 mRNA in *Xenopus *oocytes (Figs. 1 and 2) [24,29]. Efficiency refers to both the fraction of the RNA that receives poly(A) and the length of poly(A) added. To test whether these differences might be due to differences in the sequences of the zebrafish and *Xenopus *cyclin B1 mRNA 3'UTRs or instead due to differences in the effectiveness of the polyadenylation and translational machinery present in these two species' oocytes, microinjection experiments of the relevant RNAs were performed with both zebrafish and *Xenopus *oocytes. Radiolabeled zebrafish cyclin B1 3'UTR RNAs were injected into *Xenopus *oocytes and after inducing maturation the injected RNAs were isolated and analyzed for polyadenylation as in Figure 2. The zebrafish cyclin B1 3'UTR-WT RNA was polyadenylated with a modest efficiency during *Xenopus *oocyte maturation (Fig. 4A, **lanes 1–3**) that was similar to what we observed when this RNA was analyzed during zebrafish oocyte maturation (Fig. 2D, **lanes 1–3**). For the zebrafish cyclin B1 3'UTR-WT RNA, phosphoimager quantitation indicated that approximately 50% of the RNA is polyadenylated (data not shown) (Figs. 2D** lane 3 and **4A** lane 3**). In addition, the same CPE sequences important for polyadenylation of cyclin B1 3'UTR during zebrafish oocyte maturation were also important during *Xenopus *oocyte maturation (Fig. 4A** lanes 5–7**). In the converse experiment, we analyzed wild type and mutant versions of the *Xenopus *cyclin B1 3'UTR RNA during zebrafish oocyte maturation (Fig. 4B). The *Xenopus *cyclin B1 3'UTR RNA was efficiently polyadenylated (Fig. 4B** lanes 1–4**, greater than 90% of the RNA is polyadenylated as determined by phosphoimager quantitation, data not shown) during zebrafish oocyte maturation, just as it is during *Xenopus *oocyte maturation [24,29]. Furthermore, the CPE elements identified as essential for this polyadenylation during *Xenopus *oocyte maturation [24,29] were also essential for the polyadenylation of this RNA during zebrafish oocyte maturation (Fig. 4B** lanes 5–7**). Thus the differences in efficiency of cyclin B1 mRNA polyadenylation during *Xenopus *and zebrafish oocyte maturation resulted from differences between the *Xenopus *and zebrafish cyclin B1 mRNA 3'UTRs.

We also tested whether the two different cyclin B1 mRNA 3'UTRs provided different levels of translational activation to a luciferase reporter mRNA during either *Xenopus *or zebrafish oocyte maturation. The *Xenopus *cyclin B1 3'UTR enhanced translation of the luciferase reporter mRNA more effectively than the zebrafish cyclin B1 3'UTR regardless of which species' oocytes were used for the experiment (Figs. 4C and 4D, compare zebrafish WT and *Xenopus *WT in both C and D). During oocyte maturation in both species, translational stimulation was due to the same sequences within the 3'UTRs required for polyadenylation (Figs. 4C and 4D, compare AAUAAA-mut and CPE-mut to WT). With the CPE-mut reporter RNA a small amount of stimulation was still observed raising the possibility of other elements that make detectable, but minimal contributions to translation (Figs. 4C and 4D). Together these results indicate that differences in translational efficiency of cyclin B1 mRNA were due to sequence differences between the zebrafish and *Xenopus *cyclin B1 3'UTRs.

## Discussion

In this report, we examined the underlying mechanisms required for the translational activation of the zebrafish cyclin B1 mRNA during zebrafish oocyte maturation. As expected based on extensive studies in *Xenopus*, the zebrafish cyclin B1 was polyadenylated during maturation, coincident with its translational activation [23]. In addition the 3'UTR of this mRNA was sufficient to activate translation of a luciferase reporter mRNA microinjected into zebrafish oocytes induced to mature *in vitro*. Both polyadenylation and translational activation of relevant zebrafish cyclin B1 mRNAs required AAUAAA and CPE-like sequences. These data together with the analysis of cyclin B1 in mouse [31] revealed that the fundamental mechanisms underlying translational activation of the cyclin B1 mRNA in vertebrate organisms are conserved. However, despite this conservation, we found that the efficiency by which the highly conserved cyclin B1 mRNA was polyadenylated and translated during *Xenopus *and zebrafish oocyte maturation differed substantially between these two species. These differences were likely not due to differences in the polyadenylation and translational machinery between these two species oocytes, but rather to differences in the 3'UTRs of their respective cyclin B1 mRNAs. Thus, variations in 3'UTR sequences and architecture may contribute to controlling different species' needs for translational efficiency of individual mRNAs while relying on the same highly conserved protein components.

### CPE-recognition by CPEB and the zorba protein

The CPEB binds to CPE sequences in the 3'UTRs of *Xenopus *mRNAs to provide specificity for maturation-specific poly (A) addition for many maternal mRNAs [8]. CPEB proteins bind CPEs via the RRM and zinc finger domains found at their C-termini [8,32]. The zebrafish ortholog of CPEB is called zorba [33]. The cyclin B1 3'UTRs from zebrafish and *Xenopus *activate mRNA translation during oocyte maturation in both their normal and heterologous contexts, suggesting that the *Xenopus *CPEB and zebrafish zorba proteins recognize the same RNA sequences to direct polyadenylation. The amino acid sequences of the *Xenopus *CPEB and zorba protein share 61% identity over the entire protein and 91% identity over the RRM and zinc finger domains [33]. This high degree of conservation is entirely consistent with the functional comparison of the *Xenopus *and zebrafish cyclin B1 3'UTRs during both species' oocyte maturation processes reported here. The UUUUAAAG and UUUU sequences identified in the ZF cyclin B1 3' UTR suggest variant non-consensus CPEs, although we have not yet tested direct interaction with either CPEB or Zorba.

### Structural and functional differences in the 3'UTRs of the cyclin B1 mRNAs

**Figure 5:**
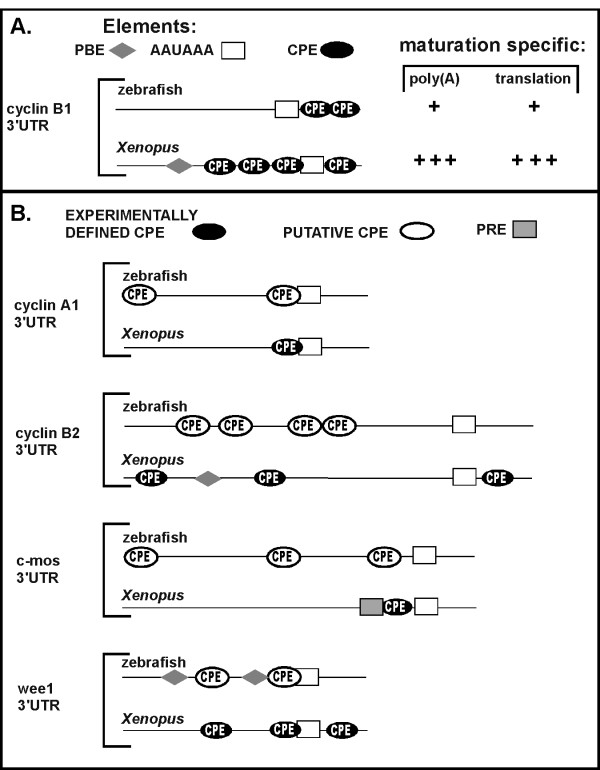
**3'UTR orthologs from zebrafish and *Xenopus *contain similar sequence elements but different architectures**. (**A**) Schematic diagram of the zebrafish and *Xenopus *cyclin B1 3'UTRs depicting the RNA sequence elements that affect maturation-specific poly (A) addition and translation. The hexanucleotide sequences AAUAAA or AUUAAA, PBE: pumilio binding elements UGUA(N)AUA, CPE: cytoplasmic polyadenylation elements UUUUAU, UUUUUCAU, UUUUAAU, UUUUACU. The activity of the cyclin B1 3'UTRs in directing poly (A) addition and activating translation during oocyte maturation in both zebrafish and *Xenopus *(this study) are summarized on the right. (**B**) Schematic diagram comparing the 3'UTR architecture of zebrafish and *Xenopus *mRNAs. These 3'UTRs were chosen for comparison because previous studies demonstrated that the *Xenopus *3'UTR in each pair is sufficient to direct poly (A) addition and activate translation during oocyte maturation. The following zebrafish mRNAs were used for analysis (listed as gene name, gene symbol, Accession number) cyclin A1, ccna1, accession BC095579; cyclin B1, ccnb1, NM_131513; b-actin2, bactin2, BC045879; cyclin B2, ccnb2, BC116569; wee1, wee1, BC116569; c-mos, mos, NM_205580, PRE – polyadenylation response element.

Our results indicate that structural differences between the zebrafish and *Xenopus *cyclin B1 mRNA 3'UTRs are responsible for the functional differences in each 3'UTR's ability to direct polyadenylation and activate translation of cyclin B1 mRNA. The sequences of the cyclin B1 mRNA 3'UTRs from zebrafish and *Xenopus *are quite different and exhibit little sequence identity (data not shown). And, even though both 3'UTRs contain CPEs the number and position of CPEs present in the two 3'UTRs differ (Figs. 2A, 2B and 5A). The zebrafish cyclin B1 3'UTR has two putative CPEs downstream of the AAUAAA hexanucleotide while the *Xenopus *3'UTR contains four CPEs, one downstream of the hexanucleotide and three upstream. In addition, pumilio binding elements (PBEs) present in the *Xenopus *cyclin B1 3'UTR direct translational repression in non-matured oocytes [34,35]. The 3'UTR of the zebrafish cyclin B1 mRNA lacks any obvious putative PBEs. Thus the number of CPEs present and their position relative to AAUAAA may dictate the efficiency of poly (A) addition and translation directed from a particular 3'UTR. This observation is consistent with the results of a recent mutational analysis of CPE function in *Xenopus *[26]. Similar differences in 3'UTR architecture are also observed comparing the 3'UTRs from other *Xenopus *mRNAs known to be polyadenylated during oocyte maturation to their zebrafish orthologs (Fig. 5B). Putative CPEs are present in the zebrafish 3'UTRs as predicted, but the arrangement and number of putative CPEs and the presence of putative PBEs differ with each pair of 3'UTR orthologs. Based on our analysis of cyclin B1 mRNA, we predict that these differences will lead to differences in the efficiency of poly (A) addition and translation for each mRNA, potentially as predicted by Pique etal 2008 [26]. This regulatory strategy allows for individualized modes of translation to ensure that specific proteins are synthesized at the proper levels to meet the unique demands of each species of developing embryos. Interestingly, the zebrafish cyclin B1 mRNA is localized to the animal pole of oocytes and eggs. This localization confines the mRNA to the embryo blastodisc where synthesis of cyclin B1 protein directs cell division during development. In contrast, the cyclin B1 mRNA in *Xenopus *is not localized. The large size of *Xenopus *eggs may require higher concentrations of cyclin B1 protein to drive early cell divisions. Such a need for producing higher concentrations of cyclin B1 protein could explain the observed differences in translational efficiency of the *Xenopus *cyclin B1 3'UTR compared to the zebrafish cyclin B1 3'UTR.

## Conclusion

Our data strongly suggest that the fundamental mechanisms underlying translational activation of the cyclin B1 mRNA in vertebrate organisms are conserved. However, despite this conservation, we found that the efficiency by which the highly conserved cyclin B1 mRNA was polyadenylated and translated during *Xenopus *and zebrafish oocyte maturation differed substantially between these two species. These differences were not due to differences in the polyadenylation and translational machinery between these two species oocytes, but could be attributed to the distinct 3'UTR elements of the respective cyclin B1 mRNAs. Thus, variations in 3'UTR sequences and architecture may contribute to controlling different species' needs for translational efficiency of individual mRNAs while relying on the same highly conserved trans-acting machinery.

## Methods

### Plasmids

Zebrafish mRNA 3'UTRs were generated by RT-PCR using total RNA extracted from early stage zebrafish embryos. cDNAs were synthesized using reverse transcriptase with either oligo dT 15 primer or a gene-specific reverse primer. The subsequent PCRs were performed with Tth DNA Polymerase. The following PCR primers were used for different 3'UTRs: zebrafish cyclin B1 (Accession NM_131513) forward primer 5'-TTATGCTGAAGAGACTTAACGACTGTGTGC-3', reverse primer 5'-CATGGATCCAAAACTTTAAAAAGTTTATTTGAATTCAAATGTACAAACTTGC-3'; zebrafish bactin2 (Accession BC045879) forward primer 5'-CGGACTGTTACCACTTCACGCCG-3', reverse primer 5'TGGATCCAGGATGTCTTACATGTGCAC-3'. PCR products for these wt 3'UTRs were ligated into pSTBlue-1 vector. Zebrafish cyclin B1 AAUAAA mutant and CPE mutant 3'UTRs were generated by PCR mutagenesis. To generate luciferase reporter plasmids, the BamH1 fragments of these 3'UTRs-containing pSTBlue1 plasmids were cloned into BamHI/BglII sites of pT7Luc vector [30].

The *Xenopus *cyclinB1 CPE-mutant 3'UTR was derived by PCR mutagenesis. The PCR product was digested with BamHI, ligated into the BglII/BamHI sites of pT7Luc vector. To generate pSTBlue-Xen cyclinB1 3'UTR CPE mutant plasmid, the above PCR product was BamHI digested, blunted and ligated into pSTBlue-1 vector. pT7Luc/A50, pGEM3Z/Xen CyclinB1-WT 3'UTR and pT7Luc/Xen CyclinB1 WT 3'UTR plasmids are as described [24].

### Luciferase Reporter mRNAs Synthesis

To prepare transcription templates for synthesizing luciferase reporter mRNAs, pT7Luc plasmids containing different 3'UTRs were linearized with *Bam*HI, pT7Luc vector alone was linearized with *Bgl*II, pT7Luc/A50 was linearized with *Dra*I. Linearized templates were transcribed *in vitro *using T7 RNA polymerase [36].

### Luciferase Assays

Cell extracts were prepared from 10–20 oocytes, eggs or embryos by homogenization in 1× cell culture lysis reagent (50 μl/frog oocyte, 20 μl/zebrafish oocyte) and centrifuged at 10000 g, 4°C for 10 min. The clear supernatant was analyzed in a luminometer following the addition of luciferase assay reagent.

### RT-PCR

Total RNA was extracted from luciferase reporter injected zebrafish oocytes as described previously for *Xenopus *oocytes [36]. RNA integrity was checked by agarose gel electrophoresis and the concentration determined by UV spectrophotometer. Total RNA from 1 oocyte was used to synthesize cDNAs and perform subsequent PCRs. The following primers were used for luciferase gene: forward primer 5'- GCTGTTTTTACGATCCCTTCAGG -3', reverse primer 5'-CGGTC AACTATG AAGAAGTGTTCG- 3' (498 nt product).

### Collection of Oocytes, Eggs and Embryos

Ovaries from anaesthetized female zebrafish were immersed in 60% L-15 medium (Gibco), and single fully grown oocytes isolated and cultured at 26°C. After 1 h of culture, damaged oocytes were discarded. *Xenopus *oocytes, eggs and embryos were collected as previously described [36].

### Microinjection and Oocyte Maturation

Zebrafish oocytes were injected with luciferase reporter RNAs in a volume of 1 nL (0.017 fmol) per oocyte. After 1 h of culture, some injected oocytes were cultured in 60% L-15 medium with 10 μg/ml 17α, 20β-dihydroxy-4-pregnen-3-one (Sigma) at 26°C for 4–6 h. Oocytes that became translucent after this treatment were harvested as mature oocytes. *Xenopus *oocytes were injected with 10 nL (0.5 fmol) RNA per oocyte and incubated at room temperature for 1–2 hours. Some of the injected oocytes were matured into eggs by treatment with progesterone (10 μg/ml) overnight at 18°C and matured oocytes were collected for analysis.

### RNaseH Treatment/RNA Blot Analysis

Total RNA was isolated from 10–20 zebrafish oocytes, matured oocytes or embryos using Trizol Reagent. Total RNA (10 ug combined with 1 mM EDTA in 20 μl volume) was heated at 100°C for one minute, chilled on ice and incubated for 10 min at room temperature with 1 μg of DNA oligonucleotide (5'-CCAGCTCATCTTCAGTGTAGCC-3') complimentary to positions -393 to -372 of the zebrafish cyclin B1 mRNA's 3'UTR (the poly (A) addition site is +1). To remove poly (A), 1 μg of oligo dT15 was added to each reaction. RNaseH treatments and product analysis using high resolution blot hybridization was performed as described [36]. The blots were hybridized with a radiolabeled probe generated from the zebrafish cyclin B1 3'UTR.

### Synthesis and Analysis of P^32 ^Labeled 3'UTRs

Plasmids pGEM3Z/*Xenopus *cyclinB1 WT 3'UTR, pSTBlue-1/*Xenopus *cyclinB1 3'UTR CPE mutant, pSTBlue-1/zebrafish cyclinB1 WT 3'UTR, pSTBlue-1/zebrafish cyclinB1 3'UTR CPE mutant were linearized by digestion with *XbaI*, SalI, SalI, SalI respectively. P^32 ^labeled 3'UTRs were generated by *in vitro *transcription as previously described [36]. The zf-WT and zf-CPE cyclin B1 3'UTR RNAs were 274 nucleotides in length. The Xen-WT and Xen-CPE cyclin B1 3'UTRs were 105 and 202 nucleotides in length respectively. The additional length of the Xen-CPE RNA is due to vector sequences. 20 nl of radiolabeled RNA was injected per *Xenopus *oocyte and 1 nl injected per zebrafish oocyte. RNAs from injected oocytes and mature oocytes were isolated and analyzed by 4% denaturing PAGE as previously described [36].

## Authors' contributions

YZ designed the study, performed the experiments, analyzed the experimental results and participated in writing the manuscript. MS conceived of the study and participated in writing the manuscript. All authors read and approved the final manuscript.

## References

[B1] Kuersten S, Goodwin EB (2003). The power of the 3'UTR: translational control and development. Nat Rev Genet.

[B2] Vardy L, Orr-Weaver TL (2007). Regulating translation of maternal messages: multiple repression mechanisms. Trends Cell Biol.

[B3] Colegrove-Otero LJ, Minshall N, Standart N (2005). RNA-binding proteins in early development. Critical Reviews in Biochemistry & Molecular Biology.

[B4] Vasudevan S, Seli E, Steitz JA (2006). Metazoan oocyte and early embryo development program: a progression through translation regulatory cascades. Genes Dev.

[B5] de Moor CH, Meijer H, Lissenden S (2005). Mechanisms of translational control by the 3'UTR in development and differentiation. Semin Cell Dev Biol.

[B6] Mendez R, Richter JD (2001). Translational control by CPEB: A means to the end [Review]. Nature Reviews Molecular Cell Biology.

[B7] Fox CA, Sheets MD, Wickens MP (1989). Poly(A) addition during maturation of frog oocytes: distinct nuclear and cytoplasmic activities and regulation by the sequence UUUUUAU. Genes & Development.

[B8] Hake LE, Richter JD (1994). CPEB is a specificity factor that mediates cytoplasmic polyadenylation during Xenopus oocyte maturation. Cell.

[B9] McGrew LL, Dworkin-Rastl E, Dworkin MB, Richter JD (1989). Poly(A) elongation during Xenopus oocyte maturation is required for translational recruitment and is mediated by a short sequence element. Genes & Development.

[B10] Stebbins-Boaz B, Cao Q, de Moor CH, Mendez R, Richter JD (1999). Maskin is a CPEB-associated factor that transiently interacts with elF-4E [published erratum appears in Mol Cell 2000 Apr;5(4):following 766]. Molecular Cell.

[B11] Mendez R, Murthy KGK, Ryan K, Manley JL, Richter JD (2000). Phosphorylation of CPEB by Eg2 mediates the recruitment of CPSF into an active cytoplasmic polyadenylation complex. Molecular Cell.

[B12] Barnard DC, Ryan K, Manley JL, Richter JD (2004). Symplekin and xGLD-2 are required for CPEB-mediated cytoplasmic polyadenylation. Cell.

[B13] Charlesworth A, Ridge JA, King LA, MacNicol MC, MacNicol AM (2002). A novel regulatory element determines the timing of Mos mRNA translation during Xenopus oocyte maturation. Embo J.

[B14] Charlesworth A, Cox LL, MacNicol AM (2004). Cytoplasmic polyadenylation element (CPE)- and CPE-binding protein (CPEB)-independent mechanisms regulate early class maternal mRNA translational activation in Xenopus oocytes. J Biol Chem.

[B15] Prasad CK, Mahadevan M, MacNicol MC, MacNicol AM (2008). Mos 3'UTR regulatory differences underlie species-specific temporal patterns of Mos mRNA cytoplasmic polyadenylation and translational recruitment during oocyte maturation. Mol Reprod Dev.

[B16] Dosch R, Wagner DS, Mintzer KA, Runke G, Wiemelt AP, Mullins MC (2004). Maternal control of vertebrate development before the midblastula transition: mutants from the zebrafish I. Dev Cell.

[B17] Tribulo C, Aybar MJ, Nguyen VH, Mullins MC, Mayor R (2003). Regulation of Msx genes by a Bmp gradient is essential for neural crest specification. Development.

[B18] Pelegri F (2003). Maternal factors in zebrafish development. Developmental Dynamics.

[B19] Connors SA, Tucker JA, Mullins MC (2006). Temporal and spatial action of tolloid (mini fin) and chordin to pattern tail tissues. Dev Biol.

[B20] Lyman Gingerich J, Westfall TA, Slusarski DC, Pelegri F (2005). hecate, a zebrafish maternal effect gene, affects dorsal organizer induction and intracellular calcium transient frequency. Dev Biol.

[B21] Yabe T, Ge X, Pelegri F (2007). The zebrafish maternal-effect gene cellular atoll encodes the centriolar component sas-6 and defects in its paternal function promote whole genome duplication. Dev Biol.

[B22] Kondo T, Yanagawa T, Yoshida N, Yamashita M (1997). Introduction of cyclin B induces activation of the maturation-promoting factor and breakdown of germinal vesicle in growing zebrafish oocytes unresponsive to the maturation-inducing hormone. Dev Biol.

[B23] Kondo T, Kotani T, Yamashita M (2001). Dispersion of cyclin B mRNA aggregation is coupled with translational activation of the mRNA during zebrafish oocyte maturation. Dev Biol.

[B24] Sheets MD, Fox CA, Hunt T, Woude G Vande, Wickens M (1994). The 3'-untranslated regions of c-mos and cyclin mRNAs stimulate translation by regulating cytoplasmic polyadenylation. Genes & Development.

[B25] Fox CA, Sheets MD, Wahle E, Wickens M (1992). Polyadenylation of maternal mRNA during oocyte maturation: poly(A) addition in vitro requires a regulated RNA binding activity and a poly(A) polymerase. Embo J.

[B26] Pique M, Lopez JM, Foissac S, Guigo R, Mendez R (2008). A combinatorial code for CPE-mediated translational control. Cell.

[B27] Colgan DF, Manley JL (1997). Mechanism and regulation of mRNA polyadenylation. Genes Dev.

[B28] Danckwardt S, Hentze MW, Kulozik AE (2008). 3' end mRNA processing: molecular mechanisms and implications for health and disease. Embo J.

[B29] Barkoff AF, Dickson KS, Gray NK, Wickens M (2000). Translational control of cyclin B1 mRNA during meiotic maturation: coordinated repression and cytoplasmic polyadenylation. Developmental Biology.

[B30] Gallie DR (1991). The cap and poly(A) tail function synergistically to regulate mRNA translational efficiency. Genes Dev.

[B31] Tay J, Hodgman R, Richter JD (2000). The control of cyclin B1 mRNA translation during mouse oocyte maturation. Dev Biol.

[B32] Hake LE, Mendez R, Richter JD (1998). Specificity of RNA binding by CPEB: requirement for RNA recognition motifs and a novel zinc finger. Mol Cell Biol.

[B33] Bally-Cuif L, Schatz WJ, Ho RK (1998). Characterization of the zebrafish Orb/CPEB-related RNA binding protein and localization of maternal components in the zebrafish oocyte. Mech Dev.

[B34] Nakahata S, Kotani T, Mita K, Kawasaki T, Katsu Y, Nagahama Y, Yamashita M (2003). Involvement of Xenopus Pumilio in the translational regulation that is specific to cyclin B1 mRNA during oocyte maturation. Mech Dev.

[B35] Nakahata S, Katsu Y, Mita K, Inoue K, Nagahama Y, Yamashita M (2001). Biochemical identification of Xenopus pumilio as a sequence-specific cyclin B1 mRNA-binding protein that physically interacts with a nanos homolog, Xcat-2, and a cytoplasmic polyadenylation element-binding protein. Journal of Biological Chemistry.

[B36] Fritz BR, Sheets MD (2001). Regulation of the mRNAs encoding proteins of the BMP signaling pathway during the maternal stages of Xenopus development. Developmental Biology.

